# Fibrillary Glomerulonephritis Leading to End-Stage Renal Disease in the Absence of Active or Chronic Hepatitis C Infection: Current Insights

**DOI:** 10.7759/cureus.61831

**Published:** 2024-06-06

**Authors:** Maryam Saleem, Maahin M Khan, Hassaan Iftikhar

**Affiliations:** 1 Nephrology, Ohio Valley Nephrology Associates, Owensboro, USA; 2 Nephrology, Washington University School of Medicine in St. Louis, St. Louis, USA; 3 Internal Medicine, Waterbury Hospital, Waterbury, USA; 4 Internal Medicine, Shifa College of Medicine, Shifa Tameer-e-Millat University, Islamabad, PAK; 5 Internal Medicine, St. Francis Medical Center, Trenton, USA

**Keywords:** dnajb9, end stage renal disease, hepatitis c, acute kidney injury, fibrillary glomerulonephritis

## Abstract

Fibrillary glomerulonephritis (FGN) is a rare glomerular disease with various etiologies, including idiopathic cases and associations with autoimmune diseases, neoplasms, and viral infections, such as Hepatitis C. We present a case of a patient who developed acute kidney injury (AKI) with atypical clinical features. A subsequent renal biopsy confirmed the diagnosis of FGN, with distinct immunofluorescence staining for DNAJB9. The patient tested positive for Hepatitis C antibodies with an undetectable viral load, indicating a past infection that had self-cleared. This finding prompted further investigation of the association between Hepatitis C and the development of FGN.

## Introduction

Fibrillary glomerulonephritis (FGN) is a rare glomerular disease that accounts for 0.6% of all native kidney biopsies [1]. Originally considered an idiopathic ailment, recent studies have uncovered associations in specific cases of autoimmune diseases, malignant neoplasms, or Hepatitis C virus (HCV) infection. The prognosis for this condition is guarded, as approximately half of the patients develop end-stage renal disease (ESRD) in less than five years [2]. There has been some literature on how HCV can contribute to the development of different forms of glomerulonephritis, such as cryoglobulinemic or non-cryoglobulinemic membranoproliferative glomerulonephritis [3]. We present a case of a patient who developed acute kidney injury (AKI) with atypical clinical features. A subsequent renal biopsy confirmed the diagnosis of FGN, with distinct immunofluorescence (IF) staining for DNAJB9. The patient tested positive for HCV antibodies with an undetectable viral load, indicating a past infection that had self-cleared. This finding prompted further investigation of the association between HCV and the development of FGN.

## Case presentation

A 49-year-old female with a medical history of hypertension, chronic obstructive pulmonary disease, bipolar disorder, and depression was admitted to the hospital in August 2022 because of right-sided flank pain associated with nausea and poor oral intake. Physical examination revealed no costovertebral angle or suprapubic tenderness. A kidney stone was initially suspected, but a computed tomography (CT) scan of the abdomen and pelvis did not reveal any evidence of nephrolithiasis. The patient was also found to have AKI with serum creatinine (SCr) of 4mg/dL (reference: 0.7-1.1mg/dL) and received intravenous fluids.

Regarding the renal history, the patient’s baseline SCr was 0.9mg/dL as of 2020. Between December 2021 and April 2022, the SCr increased to 1.4mg/dL, and the patient was seen by a nephrologist as an outpatient. This increase in SCr was thought to be secondary to uncontrolled hypertension due to concerns of medication non-adherence as well as nonsteroidal anti-inflammatory drug (NSAID) use. Laboratory values on admission are presented in Table 1.

**Table 1:** Laboratory tests on admission

Laboratory Test	Results	Reference Range
Serum creatinine	2.0 mg/dL	0.7-1.3 mg/dL
Serum albumin	3.2 mg/dL	3.4-5.7 mg/dL
24-hour urine protein	11000 mg	<80 mg
Urinalysis	2+ blood, 3+ protein	negative blood and protein
Serum low-density lipoprotein	200 mg/dL	<100 mg/dL

**Figure 1:**
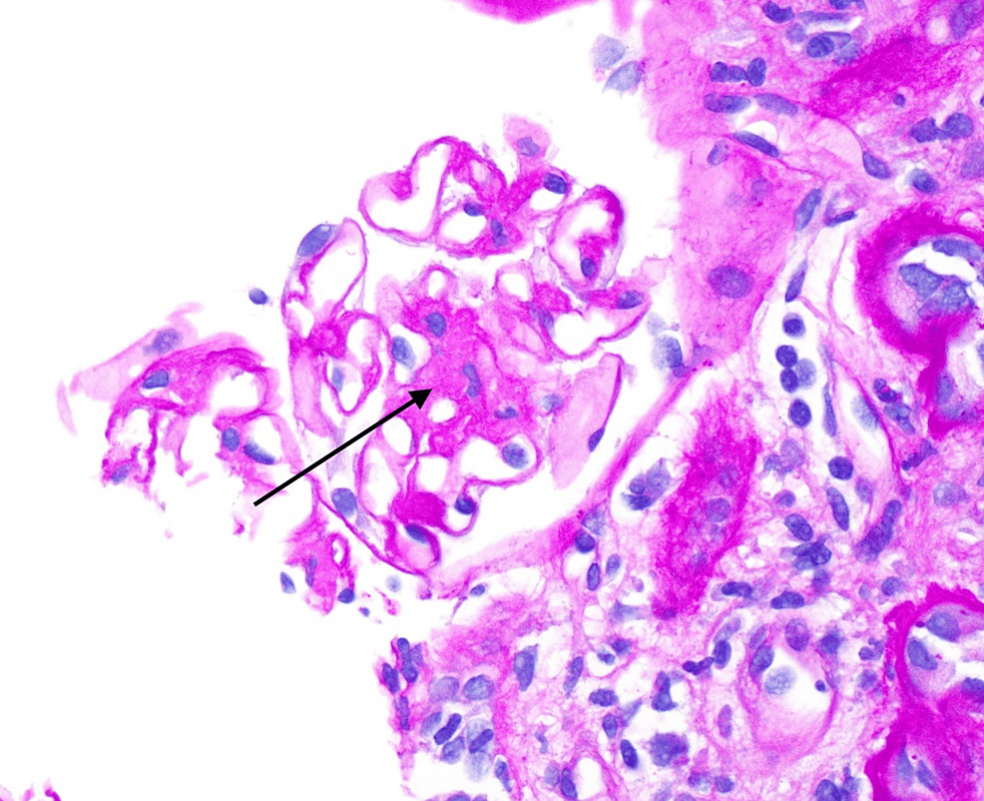
Mesangial expansion (black arrow) and capillary loop thickening on light microscopy

**Figure 2:**
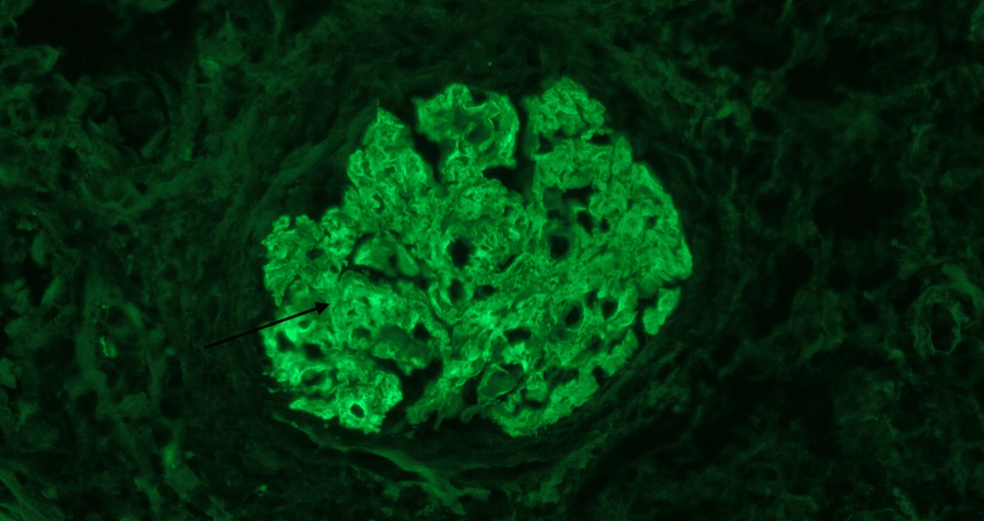
Immunofluorescence mesangial glomerular staining for IgG (black arrow)

**Figure 3:**
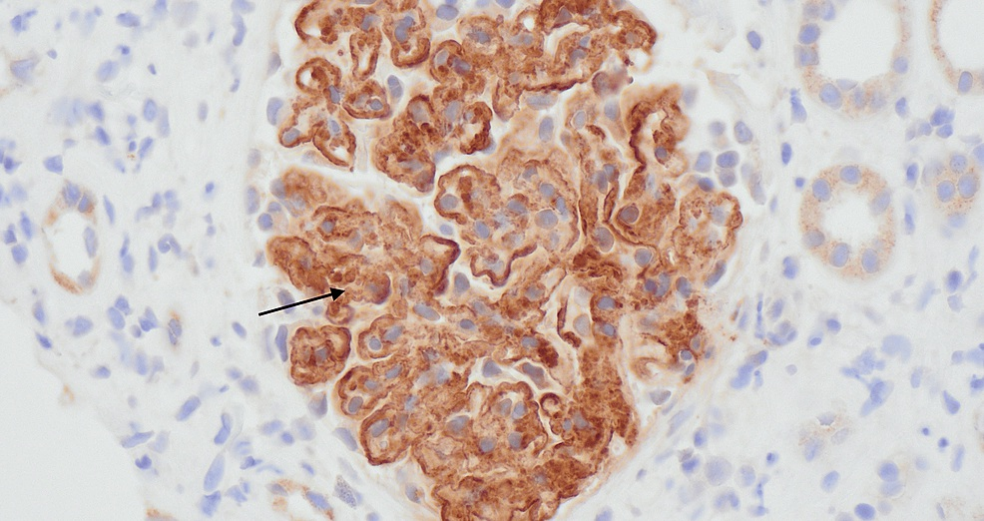
DNAJB9 stain (black arrow) on immunohistochemistry

**Figure 4:**
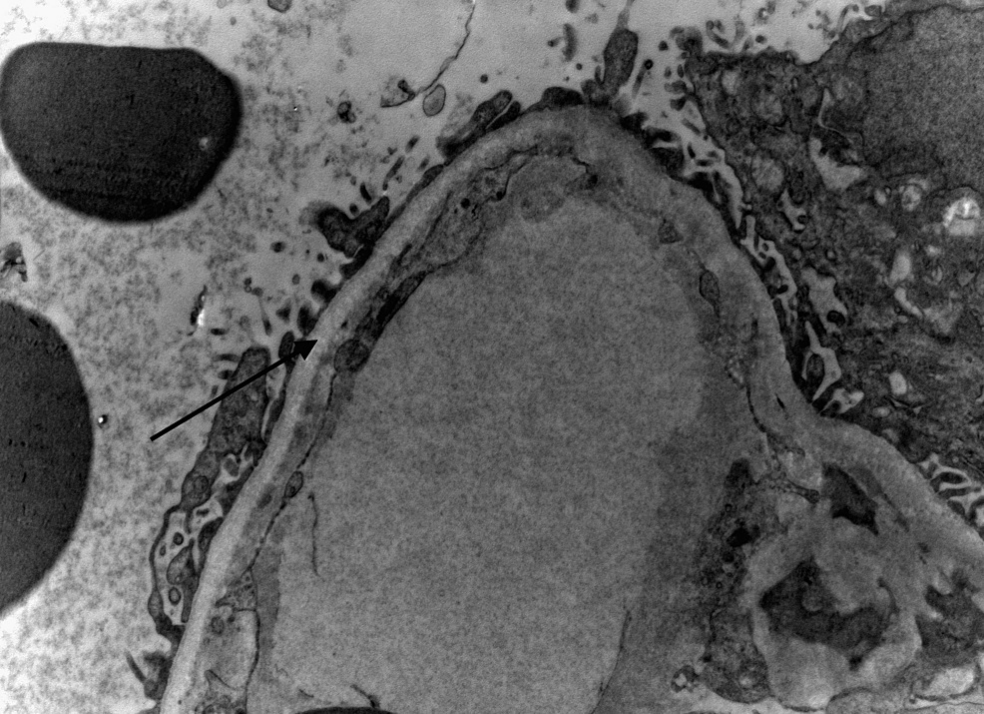
Non-branching overlapping fibrils (black arrow) in the glomerular basement membrane

On exam, she had volume overload. Due to concern for nephrotic syndrome, a serological workup was ordered and it was negative for anti-glomerular basement membrane antibody, anti-double-stranded DNA antibody (dsDNA), anti-neutrophil cytoplasmic antibody (ANCA), and Sjogren's syndrome antibodies (SS-A and SS-B). Complement levels were normal. Human immunodeficiency virus (HIV) was negative and serum protein electrophoresis and urine immunofixation were without any evidence of monoclonal protein. The patient tested negative for an age-appropriate cancer screening workup including a colonoscopy, breast mammogram and pap smear. Interestingly HCV antibody testing was positive; however, HCV polymerase chain reaction (PCR) viral load and detection test was undetectable, indicating past infection, which the patient was unaware of and had never been treated before. A kidney biopsy (as shown in Figures 1-4) revealed FGN, a positive stain for DNAJB9 on immunohistochemistry, and an IF mesangial glomerular stain for IgG. The fibril size on electron microscopy was 12 nanometers.

The underlying cause was likely HCV-related FGN, as most of the secondary causes, including other infections, malignancy/myeloma, and Lupus/Sjogren's syndrome, were ruled out based on the negative workup. Cryoglobulins were undetectable. Usually, DNAJB9 is associated with monoclonal gammopathy, however, the workup was negative. The patient's renal function continued to deteriorate, ultimately leading to dialysis initiation. The patient remained dialysis-dependent. There have been case reports of active HCV infection associated with FGN; however, very limited data are available on FGN without active HCV infection, which makes our case unique [3-6].

## Discussion

FGN is a rare glomerular disease that exhibits distinctive ultrastructural characteristics, including the presence of orderly arranged, randomly oriented fibrillar deposits of immunoglobulins [1,2,7]. According to a literature review, the average age of onset is usually the sixth decade, 66% of the patients are female, and the majority of individuals belong to the White race [7]. Although originally regarded as an idiopathic condition, recent investigations have illuminated connections between FGN and autoimmune diseases, malignant neoplasms, and HCV infection in specific cases. Instances of autoimmune diseases in patients with FGN included Crohn's disease, systemic lupus erythematosus, Graves’ disease, and idiopathic thrombocytopenic purpura. Given the occurrence of these conditions, it is advisable to screen patients with FGN for HCV, dysproteinemia, autoimmune diseases, diabetes mellitus, and malignancy [1,2,7,8]. Patients usually present with signs of renal insufficiency characterized by an elevated SCr level and nephrotic-range proteinuria, with 36% of patients fulfilling the criteria for nephrotic syndrome [7]. FGN is a pathological diagnosis, and kidney biopsy shows nonspecific findings on light microscopy, such as focal mesangial, diffuse proliferative, or membranoproliferative glomerulonephritis (with or without crescent formation) and a membranous pattern with mesangial expansion. IF reveals intense staining for IgG, particularly IgG4 and IgG1, along with the deposition of immune complexes in the glomeruli, as evidenced by the presence of C3, kappa, and lambda light chains. The lack of Congo red staining, with a few exceptions, and the distinct composition of the fibrils serve as distinguishing factors between FGN and amyloidosis. Additionally, the diameter of the fibrils and the absence of a microtubular structure with a hollow core aid in differentiating FGN from immunotactoid glomerulopathy [2,7]. A recent breakthrough in FGN diagnosis stems from the remarkable discovery of DNAJB9, a highly accurate and precise indicator of this condition. Glomerular DNAJB9 expression had a sensitivity and specificity of >98%. DNAJB9, identified as a proteomic marker through mass spectrometry, is usually detected in all cases of FGN regardless of the presence of congophilia. However, it is not found in cases of amyloidosis or in healthy individuals [9,10]. Our case strongly suggests that a history of HCV is the probable underlying cause of FGN. Recent advances have revealed that HCV can contribute to the development of different forms of glomerulonephritis, such as cryoglobulinemic or non-cryoglobulinemic membranoproliferative glomerulonephritis [3]. Membranoproliferative glomerulonephritis is the most commonly observed glomerular disease associated with HCV infection. HCV infection has also been linked to other glomerular diseases, including membranous nephropathy, focal segmental glomerulosclerosis (FSGS), fibrillary glomerulopathy, immunotactoid glomerulopathy, and IgA nephropathy [4]. HCV1 and HCV2, both genotypes, have been associated with the presence of chronic kidney disease and the development of ESRD. Although a complete understanding of how different HCV genotypes affect renal outcomes is still evolving, it is crucial to include diligent evaluation of renal function as a routine part of follow-up for individuals with HCV infection, particularly in cases of elevated serum HCV RNA levels and HCV genotype 1 or 2 infections [5]. Treatment of FGN from secondary causes should focus on treating underlying disorders, such as malignancy, autoimmune disease, monoclonal gammopathy, and infections. In individuals with idiopathic FGN, treatment depends on estimated glomerular filtration rate (GFR) and proteinuria. A conservative approach is usually considered in patients with non-nephrotic-range proteinuria and an estimated GFR >60 and comprises angiotensin blockade [6]. Unfortunately, there are no trials to guide the treatment of FGN in patients with estimated GFR <60 and nephrotic-range proteinuria. An expert opinion suggests the use of rituximab based on data from case reports [11,12]. The prognosis for FGN is guarded, with nearly half of the patients progressing to ESRD in less than five years [1].

## Conclusions

HCV infection has been associated with various forms of glomerulonephritis. Monitoring renal function is crucial in individuals with HCV infection, especially in cases of elevated serum HCV RNA levels and infections with different HCV genotypes. Given the multitude of pathologies that can affect glomeruli, it is crucial to thoroughly screen patients with HCV for any signs of glomerular involvement. Our patient did not have active HCV, as indicated by an undetectable viral load on PCR, but was positive for IgG, suggesting a previous infection that may have self-cleared. However, the patient developed ESRD from FGN, raising the question of whether FGN can still occur in individuals with cleared HCV infection. Further investigations are needed to uncover the possible mechanisms that may impact eventual treatment outcomes.
